# Effects of a functional fatty acid blend on growth performance, intestinal morphology, and serum profiles in weaned piglets

**DOI:** 10.5713/ab.22.0290

**Published:** 2022-11-14

**Authors:** Huakai Wang, Yanan Wang, Yu Zhang, Juntao Li, Yihai Mi, Yongqiang Xue, Jiaan Li, Yongxi Ma

**Affiliations:** 1 State Key Laboratory of Animal Nutrition, College of Animal Science and Technology, China Agricultural University, Beijing 100193, China; 2 CALID BIOTECH (WUHAN) CO., LTD, Wuhan 430073, China

**Keywords:** Antioxidant Capacity, Anti-inflammatory, Fatty Acid, Immune Status, Intestinal Morphology, Piglets

## Abstract

**Objective:**

The objective of this study was to determine whether dietary supplementation with a functional fatty acid blend (FA) that contains 31.4% butyric acid and 4.99% medium-chain FA improve growth performance, antioxidant capacity, immunity status, and anti-inflammatory ability in weaned piglets.

**Methods:**

One hundred and forty-four healthy piglets (Duroc×Landrace×Yorkshire) with an average body weight (BW) of 7.98±3.43 kg were randomly divided into three groups with six replicate pens and eight piglets per pen: Normal control (NC): a corn-soybean basal diet; FA1: a basal diet supplemented with 1,000 mg/kg of a functional FA; FA2: a basal diet supplemented with 2,000 mg/kg of a functional FA. The experiment lasted for 28 d. On d 14 and 28, one piglet in each pen from NC and FA2 groups was randomly selected for antioxidative index and immunoglobulins. On d 28, one piglet in each pen from NC and FA2 groups was randomly selected for intestinal morphology and inflammatory factor.

**Results:**

We observed that FA supplementation linearly increased (p<0.05) average daily gain and the final BW. There was higher (p<0.05) catalase on d 14, and immunoglobulin (Ig) A and IgM on d 28 in piglets supplemented with FA2 than in the NC group. Moreover, dietary FA2 reduced (p<0.05) crypt depth of ileum in piglets. The concentrations of tumor necrosis factor-α, interleukin (IL)-1β, IL-8, and IL-10 in jejunum were lower (p<0.05) in the FA2 group compared with the NC group.

**Conclusion:**

Therefore, the overall results suggests that the FA may help to improve gut health, antioxidant status, and immune parameters resulting in the improvement of growth performance.

## INTRODUCTION

Piglets can suffer from a series of health problems after weaning due to inadequate digestive systems, such as reduced feed intake, diarrhea, inflammation, and death in severe cases [1]. Feed supplements or additives are used to control pathogenic microorganisms and to improve feed efficiency and animal performance. Antibiotic growth promoters have been used to improve animal health and growth performance in animal production due to their better efficacy over the past decades [2]. However, the overuse of antibiotic growth promoters resulted in severe residues and was therefore banned by the European Union in 2006. As a result, natural additives that can effectively inhibit bacteria have become an important research focus [3].

Fatty acids (FA), not only contribute to the absorption of fat-soluble vitamins, but also are the main components of the body’s cells, which are particularly important for the metabolism. Manipulation of the milk FA composition via sow nutrition, or inclusion of dietary fat sources in the feed for weaned pigs, may be used as strategic tool to improve pig performance pre- and post-weaning [4]. Butyric acid, a short-chain FA produced by colonic microbes, possesses important functions in maintaining the balance of intestinal microflora and reducing the generation of pro-inflammatory cytokines, which has been widely used to improve animal growth performance in recent years [5,6]. Medium-chain fatty acids (MCFA) with 6 to 12 carbon atoms, including caproic (C6), caprylic (C8), capric (C10), and lauric (C12) acids. MCFA can be absorbed directly into the portal blood and may contribute to immediate energy for the enterocytes [7,8]. Moreover, MCFA possess antibacterial effects and may thereby reduce the amount of viral and bacterial pathogens in the feed and the risk of disease transmission [9]. Previous studies showed that organic acid blends containing lactic acid could reduce concentrations of *Salmonella* in feed and improve the growth performance in nursery pigs [10,11]. Commercial products are becoming available with proprietary blends of MCFA, as well as other ingredients. Because of the differences in response to feeding different mixtures of free FAs and MCFA, it was necessary to evaluate the products and their effects on growth performance [12]. Therefore, the objective of this study was to evaluate the effects of dietary functional fatty acid blend (FA), which contains 31.4% butyric acid and 4.99% MCFA, on growth perfor mance, antioxidant capacity, immunity status, intestinal morphology, and jejunal inflammatory factor in weaned piglets.

## MATERIALS AND METHODS

The experiments were conducted at the FengNing Swine Research Unit of China Agricultural University (Chengdejiuyun Agricultural and Livestock Co., Ltd., Hebei, China) and all animal procedures were approved by the animal ethical committee of China Agricultural University (Beijing, China; No. AW90602202-1-2). The composition of the FA used in this research is shown in Table 1 and provided by CALID BIOTECH (WUHAN) CO., LTD. (Wuhan, China).

### Animals and experimental design

A total of 144 ([Duroc×Landrace×Yorkshire], with an average weight of 7.97±3.43 kg) weaned piglets were randomly divided into three treatments with six replicates (8 piglets per replicate pen, 4 barrows, and 4 gilts per pen) per treatment. Based on the results of our previous experiments on broilers and ducks (unpublished data), we chose two concentrations (1,000 mg/kg and 2,000 mg/kg) of FA. The three treatments are a corn-soybean basal diet (NC), the NC with 1,000 mg/kg FA supplementation (FA1), and the NC with 2,000 mg/kg FA supplementation (FA2). The corn-soybean basal diets, shown in Table 2, were formulated to meet the recommended requirements [13]. All piglets were kept in experimental pens with relatively constant temperature (26°C to 28°C) and humidity (60% to 70%). The piglets had *ad libitum* access to feed and water during the experimental period (28 d).

### Growth performance

Piglets were weighed at 0, 14, and 28 d of experiment individually to calculate the average daily gain (ADG). Feed intake was recorded during the experimental period for each pen every two weeks to calculate the average daily feed intake (ADFI). The ADG:ADFI (G:F) for each pen was calculated subsequently.

### Sample collection

On the morning of d 14 and 28, one piglet in each pen from NC and FA2 groups was randomly selected for the collection of serum. Serum samples were collected from the external jugular vein in 10-mL vacuum tubes and were separated by centrifugation (3,000×*g* for 15 min at 4°C) and stored at −20°C until analysis. One piglet from each pen from NC and FA2 groups was randomly selected to slaughter at the end of this experiment. The duodenum, jejunum, and ileum were fixed in 4% paraformaldehyde for 24 h at 25°C for morphological observation. Moreover, an approximately 2 cm length of the jejunum was collected and stored at −80°C for inflammatory factor analysis.

### Analysis of antioxidative index and immunoglobulins

Serum total antioxidant capacity (T-AOC), superoxide dismutase (SOD), malonaldehyde (MDA), glutathione peroxidase (GSH-Px), catalase (CAT), immunoglobulin (Ig) A, IgG, and IgM were analyzed by enzyme-linked immunosorbent assay (ELISA) according to the instructions of manufacturers (Nanjing Jiancheng Bioengineering Institute, Nanjing, China). Briefly, the T-AOC was detected using a spectrophotometer (LengGuang SFZ1606017568, Shanghai, China) at 520 nm measuring the development of stable and colored chelates. The SOD activity was quantified with the xanthine superoxide anion radical product and oxidase reaction system, and the MDA concentration was quantified with the thiobarbituric acid reactive substances method. The activity of GSH-Px was quantified by measuring the consumption rate of reduced glutathione in the enzymatic reaction. The CAT activity was detected at 240 nm based on the consumption of H_2_O_2_. According to the optical density values of standards, IgA, IgG, and IgM were measured with UV-2401PC at 700 nm, 340 nm, and 340 nm, respectively (UV–vis recording spectrophotometer; SHIMADZU Corporation, Kyoto, Japan). The immunoglobulin concentration of the samples (g/L) was calculated with the standard curve.

### Intestinal morphology

The fixed samples of the small intestine were dehydrated, cleared, and then embedded in paraffin. The samples were sectioned at 5 μm and stained with hematoxylin and eosin (H&E) following the methods of Chwen et al [14]. Villus height and crypt depth were observed by a microscope analyzed by Leica Imaging Systems (version 1; Leica Imaging Systems Ltd., Cambridge, UK).

### Jejunal inflammatory factor

Jejunal tumor necrosis factor-α (TNF-α), interferon-γ (IFN-γ), interleukin (IL) -1β, IL-6, IL-8, and IL-10 were determined by ELISA following the methods of manufacturers (Nanjing Jiancheng Bioengineering Institute, China). Following the kit manufacturer’s operating instructions, the levels of TNF-α, IFN-γ, IL-1β, IL-6, IL-8, and IL-10 were calculated with the standard curve and were expressed as nanograms per liter.

### Statistical analysis

The data was analyzed as a randomized complete block design using the general linear model procedure of SAS 9.2 (SAS Institute, Cary, NC, USA). The statistical model included the diets as fixed effect, and the blocks as random effects. The data were calculated for each pen to analyze growth performance, and for individual piglets to analyze other parameters. Significant differences were considered at p<0.05, and 0.05< p<0.10 was considered as trending to significance.

## RESULTS

### Growth performance

The effects of dietary supplementation with FA on growth performance in weaned piglets are shown in Table 3. Dietary functional FA blend did not affect the ADFI and G:F, while FA supplementation increased (p<0.05) linearly the ADG and the final body weight (BW) compared with the NC group.

### Antioxidant capacity in serum

As shown in Table 4, dietary FA2 did not affect the serum T-AOC, SOD, MDA, GSH-Px. However, higher serum CAT level was observed (p<0.05) in the FA2 group compared with the NC group.

### Immunity status in serum

As shown in Table 5, dietary FA2 increased (p<0.05) the serum IgA and IgM concentrations on d 28, while having no effect on serum concentration of IgG.

### Intestinal morphology

Table 6 shows the effects of FA on villus height, crypt depth, and villus height:crypt depth ratio in weaned piglets. Dietary FA2 reduced (p<0.05) crypt depth in the ileum compared with the NC group, while it did not affect other intestinal morphology parameters.

### Jejunal inflammatory factor

As shown in Table 7, supplementation with FA2 reduced (p<0.05) the levels of TNF-α, IL-1β, IL-8, and IL-10 compared with the NC group.

## DISCUSSION

To improve postweaning health, minimize the postweaning growth lag, and increase feed intake, newly weaned piglets are often offered diets supplemented with additives that have no pollution to the environment, and strong resistance to disease properties. Dietary supplementation with sodium butyrate was shown to improve the growth performance of nursing piglets, weaned piglets, and growing-finishing pigs [15,16]. In the present study, the results showed that dietary supplementation with FA increased the ADG compared with the NC group, which may be attributed to the regulation of gut bacterial community structures by butyrate [5]. In addition, sodium butyrate, an important energy source for intestinal epithelial cells, can reduce the waste of starch and amino acids in the diets, consequently resulting in more nutrients to be used for growth [17]. However, Biagi et al [18] reported that dietary supplementation with sodium butyrate had no effect on the growth performance of piglets, which was not in agreement with our results. The discrepancies may be due to the difference of FA blend. Moreover, MCFA has been shown to effectively improve the absorption of nutrients and lipid metabolism of piglets to meet the energy demand of weaned piglets, and then improve the growth performance of weaned piglets [8]. In the current experiment, supplementation with FA2 had a better effect on the growth performance compared with the FA1 group, thus we selected the FA2 to conduct the next study.

Weaning stress can increase the formation of reactive ox ygen species (ROS) and lead to oxidative stress and damaged tissue [19]. The T-AOC reflects the total scavenging capacity of ROS:nitric oxide synthase (ROS:NOS) to a certain extent. The antioxidant enzymes, including SOD, GSH-Px, and CAT, are the essential components of oxidative stress defense systems [5]. In the current study, we evaluated these indicators related to the antioxidant system and MDA, a marker for measuring the degree of oxidative stress. Dietary supplementation with FA2 increased the serum CAT activity compared with the NC group, indicating FA supplementation might have boosted the oxidative stress defense system. Zhang et al [20] reported that dietary sodium butyrate decreased MDA concentration and increased SOD activity of serum in chicken. Furthermore, dietary supplementation of sodium butyrate was shown to increase GSH-Px activity and decrease MDA concentration in pre-weaned dairy calves [21]. In general, these results all showed that butyrate could improve antioxidant ability in animals. Nuclear factor E2-related factor-2 (Nrf2) is a transcription factor that orchestrates the expression of a battery of anti-oxidant and detoxification genes under both basal and stress conditions [22]. Medium-chain fatty acids, primarily lauric acid, have been shown to enhance antioxidant capacity in piglets and laying hens [3,23]. Our results showed dietary FA supplementation contributes to enhancing the antioxidant capacity in piglets.

Immunoglobulin A, IgG, and IgM are 3 important anti bodies of the immune function in piglets, which can protect animals against a variety of pathogens and viruses and improve animals immunity status [24]. A previous study found that butyrate as a histone deacetylase inhibitor can inhibit the activation of NF-κB by entering the cells and then down-regulate the expression of TNF-α, IL-1β, IL-6 [25]. Fang et al [26] reported that sodium butyrate can increase the serum IgG concentration by increasing the number of IgA+ cells in the jejunum of piglets. In the current study, supplementation with FA2 increased serum IgM concentration on d 28, which is consistent with the previous studies [25,26]. However, Liu et al [21] found that dietary sodium butyrate did not affect the serum concentrations of any of the 3 antibodies of calves, this may be due to the animal species used or methods of butyrate supplementation. Moreover, studies *in vivo* and *ex vivo* found that anti-microbial action of MCFA on *E. coli* and the improvement of MCFA on IgA in piglets [27]. Taken together, FA exerted an immunomodulatory role.

Intestinal morphological indicators include villus height, crypt depth, and villus height:crypt depth, which reflect intestinal health status. Higher villus height and villus height:crypt depth and lower crypt depth facilitate digestion and absorption of nutrients [28]. In the present study, supplementation with FA reduced the crypt depth of the ileum in piglets. This observation is similar to a previous study [29], which reported that the combined use of sodium butyrate and reduced antibiotics reduced the crypt depth of the duodenum, jejunum, and ileum. Dietary MCFA supplementation promoted the maturation of intestinal epithelial cells and villi and increased absorption of nutrients in piglets by increasing the number of goblet cells [30]. Furthermore, MCFA could protect the health of the intestinal barrier by increasing the expression of claudin-1, occluding, and ZO-1 proteins, thus effectively preventing bacterial endotoxin and toxic macromolecules from entering the body [30]. Thus, FA ameliorated gut morphology as evidenced by decreased crypt depth in the current study.

Immune stimulation after weaning results in the pro duction of pro-inflammatory factors, which suppresses the growth of piglets. The production of pro-inflammatory factors including TNF-α, IL-1β, and IL-8 initiates an array of inflammatory mediators and thus leading to the inflammatory reaction. Our study showed that supplementation with FA reduced the concentrations of TNF-α, IL-1β, and IL-8, which was partly consistent with the results of Cui et al [30] who found that MCFA reduced the level of IL-6 in the ileum when compared with the control group. Previous studies also showed that MCFA could significantly reduce the level of proinflammatory cytokines, such as IL-6 and IL-1, and relieve the inflammation of colitis in rats [31,32]. In addition, butyrate might also play a part in the anti-inflammatory process. Suppressor of cytokine signaling 3 (SOCS3) is a modulator of macrophage polarization, the beneficial role of it has been documented in restricting inflammatory responses [33]. Weber and Kerr reported that butyrate exerts anti-inflammatory activity by increasing the expression of SOCS3 and regulates the production of IL-10 and IFN-γ via a cAMP-dependent pathway [34]. Interleukin-10 is an anti-inflammatory cytokine that plays an important role in inhibiting Th1 cells responses [35]. Therefore, reduced IL-10 concentration in the FA2 group showed a lower inflammatory response in the FA2 group.

In summary, the current study showed that supplementa tion with FA may help to improve the growth performance by improving immunity, antioxidant status, and intestinal health.

## Figures and Tables

**Table 1:** The composition of the functional fatty acid blend

Items	g/100 g
Butyric acid	31.40
Caprylic acid	2.77
Decylic acid	2.17
Lauric acid	0.05
Myristic acid	0.12
Pentadecylic acid	0.02
Palmitic acid	0.51
Hexadecanoic acid	0.15
Heptadecanoic acid	0.02
Stearic acid	0.13
Oleic acid	0.42
α-linolenic acid	0.29
Linoleic acid	0.05
Eicosenoic acid	0.04
Eicosapentaenoic acid	0.21
Docosahexaenoic acid	0.38
n-6 PUFAs	0.29
n-3 PUFAs	0.64

PUFA, polyunsaturated fatty acid.

**Table 2:** Ingredient and nutrient level of the basal diets (as-fed basis, %)

Items	Period 1 (Day 0 to 14)	Period 2 Day 14 to 28)
Ingredients		
Corn	65.23	70.15
Soybean meal (43%)	10.00	12.00
Soy protein isolate	5.00	3.00
Soy protein concentrate	3.00	2.00
Whey powder	8.00	5.00
Fish meal	4.00	4.00
Soybean oil	1.50	1.00
Dicalcium phosphate	0.82	0.42
Limestone	0.85	0.90
Salt	0.30	0.30
L-lysine HCl (78.8%)	0.50	0.47
DL-Methionine (98%)	0.09	0.07
L-Threonine (98%)	0.17	0.15
L-Tryptophan (98%)	0.04	0.04
Premix^1)^	0.50	0.50
Nutrient levels^2)^		
Digestible energy (MJ/kg)	14.85	14.74
Crude protein (%)	20.09	18.62
SID^3)^ Lysine (%)	1.35	1.23
SID Methionine (%)	0.39	0.36
SID Threonine (%)	0.79	0.73
SID Tryptophan (%)	0.22	0.20
Calcium (%)	0.80	0.70
Available phosphorus (%)	0.40	0.33

1) The premix provided the following per kilogram of diet: vitamin A, 12,000 IU; vitamin D_3_, 2,000 IU; vitamin E, 30 IU; vitamin K_3_, 3 mg; vitamin B_1_, 3 mg; vitamin B_2_, 10 mg; vitamin B_6_, 6 mg; vitamin B_12_, 24 μg; nicotinic acid, 30 mg; D-pantothenic acid, 30 mg; folic acid, 2 mg; biotin, 0.3 mg; choline chloride, 600 mg; Fe, 120 mg; Cu, 10 mg; Mn, 35 mg; Zn, 120 mg; I, 0.3 mg; Se, 0.3 mg.

2) Nutrient levels are calculated according to NRC [13].

3) SID, standardized ileal digestible.

**Table 3:** Effects of dietary functional fatty acid blend (FA) on performances of piglets

Items	Treatment^1)^			SEM	p-value	
	
	NC	FA1	FA2		Linear	Quadratic
Initial body weight (kg)	8.00	7.84	8.06	0.15	0.88	0.57
Final body weight (kg)	16.89	17.66	18.57	0.31	<0.05	0.91
Day 0 to 14						
ADG (g)	272	305	319	8.32	<0.05	0.58
ADFI (g)	484	547	507	28.06	0.75	0.42
G:F	0.57	0.57	0.63	0.04	0.11	0.37
Day 14 to 28						
ADG (g)	363	396	433	9.10	<0.05	0.91
ADFI (g)	701	775	776	31.00	0.90	0.17
G:F	0.54	0.52	0.56	0.06	0.28	0.28
Day 0–28						
ADG (g)	318	350	376	7.74	<0.05	0.81
ADFI (g)	592	661	641	28.48	0.90	0.27
G:F	0.56	0.54	0.58	0.04	0.19	0.19

1) NC, piglets in the control group fed a basal diet; FA1, piglets in FA1 group fed a basal diet supplemented with 1,000 mg/kg functional fatty acid blend; FA2, piglets in FA2 group fed a basal diet supplemented with 2,000 mg/kg functional fatty acid blend.

SEM, standard error of the mean; ADG, average daily gain; ADFI, average daily feed intake; G:F, ADG:ADFI.

**Table 4:** Effects of dietary functional fatty acid blend (FA) on serum antioxidant capacity in piglets

Items	Treatment^1)^		SEM	p-value

	NC	FA2		
Day 14				
T-AOC (U/mL)	8.55	9.34	0.29	0.18
SOD (U/mL)	52.87	54.67	0.72	0.23
MDA (nmol/mL)	1.52	1.63	0.03	0.10
GSH-Px (μmol/L)	8.64	8.14	0.59	0.70
CAT (U/mL)	10.04	11.63	0.90	<0.05
Day 28				
T-AOC (U/mL)	9.33	9.04	0.25	0.59
SOD (U/mL)	51.06	51.90	1.30	0.77
MDA (nmol/mL)	1.60	1.68	0.03	0.20
GSH-Px (μmol/L)	14.86	13.69	0.71	0.44
CAT (U/mL)	13.76	14.82	0.54	0.37

1) NC, piglets in the control group fed a basal diet; FA2, piglets in FA2 group fed a basal diet supplemented with 2,000 mg/kg functional fatty acid blend.

SEM, standard error of the mean; T-AOC, Total antioxidant capacity; SOD, Superoxide dismutase; MDA, Malondialdehyde; GSH-Px, glutathione peroxidase; CAT, Catalase.

**Table 5:** Effects of dietary functional fatty acid blend (FA) on serum immunity status in piglets

Items	Treatment^1)^		SEM	p-value

	NC	FA2		
Day 14				
IgA (μg/mL)	19.12	20.84	0.73	0.26
IgG (mg/mL)	10.08	10.14	0.32	0.93
IgM (μg/mL)	8.10	8.29	0.15	0.56
Day 28				
IgA (μg/mL)	15.51	19.06	0.73	<0.05
IgG (mg/mL)	9.44	10.20	0.23	0.10
IgM (μg/mL)	6.78	7.68	0.20	<0.05

1) NC, piglets in the control group fed a basal diet; FA2, piglets in FA2 group fed a basal diet supplemented with 2,000 mg/kg functional fatty acid blend.

SEM, standard error of the mean; IgA, immunoglobulin A; IgG, immunoglobulin G; IgM, immunoglobulin M.

**Table 6:** Effects of dietary functional fatty acid blend (FA) on small intestinal morphology in piglets

Items	Treatment^1)^		SEM	p-value

	NC	FA2		
Duodenum				
Villus height (μm)	341.04	349.56	20.39	0.83
Crypt depth (μm)	558.81	582.50	26.12	0.64
Villus height:crypt depth	0.62	0.62	0.05	0.97
Jejunum				
Villus height (μm)	327.70	314.84	16.34	0.37
Crypt depth (μm)	362.82	347.40	13.71	0.56
Villus height:crypt depth	0.91	0.88	0.05	0.54
Ileum				
Villus height (μm)	356.35	337.06	14.83	0.50
Crypt depth (μm)	357.91	296.06	17.05	<0.05
Villus height:crypt depth	1.01	1.08	0.04	0.31

1) NC, piglets in the control group fed a basal diet; FA2, piglets in FA2 group fed a basal diet supplemented with 2,000 mg/kg functional fatty acid blend.

SEM, standard error of the mean.

**Table 7:** Effects of dietary functional fatty acid blend (FA) on jejunal inflammatory factor levels in piglets

Items	Treatment^1)^		SEM	p-value

	NC	FA2		
TNF-α (ng/g)	65.36	51.66	3.61	<0.05
IFN-γ (pg/mg)	144.57	141.20	1.49	0.31
IL-1β (ng/g)	114.52	95.87	4.81	<0.05
IL-6 (ng/g)	40.05	34.64	2.53	0.31
IL-8 (ng/g)	100.58	82.53	4.54	<0.05
IL-10 (ng/g)	25.00	19.13	1.38	<0.05

1) NC, piglets in the control group fed a basal diet; FA2, piglets in FA2 group fed a basal diet supplemented with 2,000 mg/kg functional fatty acid blend.

SEM, standard error of the mean; TNF-α, tumor necrosis factor-α; INF-γ, interferon-γ; IL-1β, interleukin-1β; IL-6, interleukin-6; IL-8, interleukin-8; IL-10, interleukin-10.
